# Neuromodulation of Synaptic Transmission in the Main Olfactory Bulb

**DOI:** 10.3390/ijerph15102194

**Published:** 2018-10-08

**Authors:** John D. Harvey, Thomas Heinbockel

**Affiliations:** Department of Anatomy, Howard University College of Medicine, 520 W Street, NW, Washington, DC 20059, USA; harj50@hotmail.com

**Keywords:** acetylcholine, brain, cannabinoid, dopamine, serotonin, noradrenaline, central nervous system, electrophysiology, neuroscience, sensory biology

## Abstract

A major step in our understanding of brain function is to determine how neural circuits are altered in their function by signaling molecules or neuromodulators. Neuromodulation is the neurochemical process that modifies the computations performed by a neuron or network based on changing the functional needs or behavioral state of the subject. These modulations have the effect of altering the responsivity to synaptic inputs. Early sensory processing areas, such as the main olfactory bulb, provide an accessible window for investigating how neuromodulation regulates the functional states of neural networks and influences how we process sensory information. Olfaction is an attractive model system in this regard because of its relative simplicity and because it links primary olfactory sensory neurons to higher olfactory and associational networks. Likewise, centrifugal fibers from higher order brain centers target neurons in the main olfactory bulb to regulate synaptic processing. The neuromodulatory systems that provide regulatory inputs and play important roles in olfactory sensory processing and behaviors include the endocannabinoid system, the dopaminergic system, the cholinergic system, the noradrenergic system and the serotonergic system. Here, we present a brief survey of neuromodulation of olfactory signals in the main olfactory bulb with an emphasis on the endocannabinoid system.

## 1. Introduction

Neuromodulation is a neurochemical process that modifies the computations performed by a neuron or network based on changing functional needs or behavior state of the subject [1]. This process modifies synapses and influences how they respond to incoming signals [1,2]. The modulation of synaptic activity by local network cells such as interneurons is called intrinsic neuromodulation, while modulation by inputs that originate from networks outside the local network is called extrinsic neuromodulation [2].

The olfactory system functions in the detection of odors, which influences food selection through the interplay of odor and taste signals. However, olfaction also serves reproductive and neuroendocrine functions and plays roles in memory, aggression, emotion, social organization, and recognition of prey and predators [3]. 

The early sensory processing areas for olfaction, such as the main olfactory bulb, provide an accessible window for examining the role of neuromodulation in the processing of sensory information. Olfaction is an attractive model system in this regard because of its relative simplicity and because it links primary olfactory sensory neurons to higher olfactory and associational networks.

Some systems outside the olfactory system provide regulatory inputs important to olfactory sensory processing and behaviors, i.e., they provide extrinsic neuromodulation to the olfactory bulb, including dopaminergic, cholinergic, noradrenergic and serotonergic systems. These extrinsic systems have been the focus of recent work on the regulation of olfactory processing in the main olfactory bulb as discussed below. Here we present a brief survey of intrinsic and extrinsic neuromodulation in the olfactory bulb by several neurotransmitter systems with a heavy focus on a relatively novel signaling system, the endocannabinoid system. 

## 2. Overview of the Olfactory System

The first step in olfaction is the binding of odorant molecules to chemosensitive cilia of the bipolar olfactory receptor neurons. There are about 20–30 cilia that project from the dendritic end of each olfactory receptor neuron and spread out across the surface of the nasal epithelium, within the layer of mucous covering the epithelium. The volatile air-borne odorant molecules enter the nasal cavity, either by way of inspired air from the external environment, or from the oral cavity having been released from ingested food substances [4].

The odorant molecule binds to the 7-helix transmembrane G-protein coupled olfactory receptor in the plasma membrane of a cilium, resulting in an increase in intraciliary cyclic AMP, and the opening of cAMP-gated cation channels. Calcium influx through these channels causes Ca^2+^-gated chloride channels to open. An efflux of chloride ions then occurs, and this has the unusual effect of causing additional depolarization. This is a unique signal amplification mechanism found only in olfactory receptors. Those depolarizing receptor potentials of the olfactory receptor neurons that are large enough to reach threshold, trigger trains of action potentials that travel in bundles of thin axons (cranial nerve I, CN I), pass through small holes in the cribriform plate of the ethmoid bone, and terminate in spherical synaptic zones called glomeruli located in the ipsilateral main olfactory bulb [4].

The particular odorant detected by an olfactory receptor neuron is determined by the olfactory receptor molecule that it expresses from among the over 300 corresponding genes found in humans (~1100 in rodents). There are tens of thousands of olfactory receptor neurons that express the same olfactory receptor protein, and, remarkably, they all find their way to the same one or two glomeruli in the main olfactory bulb to make their synaptic contacts. In contrast, there are only relatively few output neurons (mainly mitral cells but also tufted cells), whose dendrites receive and subsequently transmit the olfactory signals to higher centers for central processing. This system of sorting and convergence in the olfactory system results in a thousand-to-one convergence from receptors to mitral cells, thereby enhancing the sensitivity of the system. The routing of olfactory receptor neurons to particular glomeruli is important for the discrimination of odorants [4].

Axons of the main olfactory bulb’s output neurons, mainly mitral cells, travel in the lateral olfactory tract to enter the base of the brain, where they distribute the olfactory signals to one or more of the cortical olfactory centers, namely the piriform cortex, the anterior parahippocampal cortex (entorhinal cortex), and the amygdala, all on the ipsilateral side, for further processing. This routing of sensory information directly from the olfactory bulb to cortical centers bypasses the thalamus, unlike the routing in other sensory systems [4,5].

The processing of social chemical stimuli or semiochemical signals is an essential function of the olfactory system for most mammals. These chemicals differ from general odorants in that they mediate the physiologic aspects of mating and aggression. These sensory signals are routed differently, that is, to the accessory olfactory bulb rather than to the main olfactory bulb, in a parallel pathway called the vomeronasal system [5].

## 3. Organization of the Main Olfactory Bulb 

**Figure 1:**
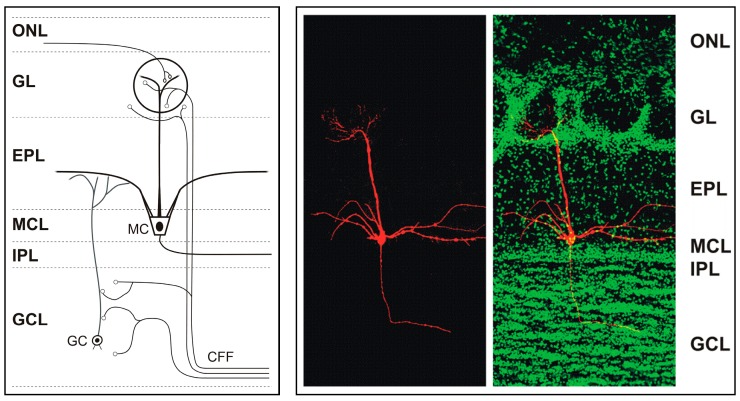
**Left:** Simplified diagram of the main olfactory bulb to illustrate its different layers, mitral cells (MCs), granule cells (GCs), and centrifugal fibers (CFF). The olfactory nerve layer (ONL) contains the axons of olfactory receptor neurons, which synapse in the spheroidal glomeruli on MCs, and other neurons. For clarity, glomerular layer (GL) cell types have been omitted from the diagram. The dendrites of the mitral cells pass from the GL through the external plexiform layer (EPL) into the mitral cell layer (MCL) which contains the cell bodies of mitral cells and many granule cells. The internal plexiform layer (IPL) contains the axons of the principal output cells (mitral cells and tufted cells) as they course to cortical areas. The granule cell layer (GCL) contains most of the granule cells as well as other inhibitory cells. CFF bring modulatory feedback signals from cortical areas, to synapse in the glomerular layer and granule cell layer. EPL, IPL—external, internal plexiform layer. **Right:** Adult mouse main olfactory bulb section with a single mitral cell intracellularly filled with biocytin (red) and nuclei stained with counterstain Sytox Green (green). The mitral cell soma is located in the MCL. One apical dendrite reaches into one glomerulus and several lateral dendrites span the main olfactory bulb. Modified from [3].

The glomerular layer of the main olfactory bulb contains 1600 to 1800 glomeruli-spheroidal structures in which olfactory receptor neurons form synapses with main olfactory bulb neurons. It is the first center where odor signals are processed and modulated, and it demonstrates a complexity of synapses not only between olfactory receptor neurons and main olfactory bulb neurons but also between main olfactory bulb neurons [4]. Mitral and tufted cells transmit olfactory information to higher centers and to other brain systems (Figure 1). Each mitral or tufted cell makes direct synaptic contact with olfactory receptor neurons as well as with local interneurons such as granule cells [6,7,8]. Mitral and tufted cells are excitatory and glutamatergic. They release glutamate at their axon terminals, apical dendrites and lateral dendrites where they form dendrodendritic synapses with granule cells. Mitral cells can be distinguished from tufted cells by their location and membrane potential bistability. Mitral cells can also be distinguished from the granule cells in the mitral cell layer due to the smaller size and higher resistance (>1 GΏ usually) of the latter [9,10,11]. 

The granule cell population in the main olfactory bulb constitutes the largest collection of GABAergic inhibitory interneurons that modulate the output of the main olfactory bulb to higher brain centers [12]. The main olfactory bulb receives abundant modulatory input from other brain areas through centrifugal fibers (CFFs) that target main olfactory bulb neurons [13]. Centrifugal fiber input is relevant for experience-dependent modulation of the main olfactory bulb, reviewed in [13]. Centrifugal projections to the main olfactory bulb originate in several cortical and hippocampal areas [14,15,16,17], mostly passing through the anterior olfactory nucleus and the anterior commissure, and very little through the lateral olfactory tract [13]. These projections arise in the locus coeruleus (noradrenergic), the horizontal limb of the diagonal band of Broca (cholinergic), and the raphe nucleus (serotonergic) [14,15,16,17]. 

## 4. Organization of the Glomerular Layer 

**Figure 2:**
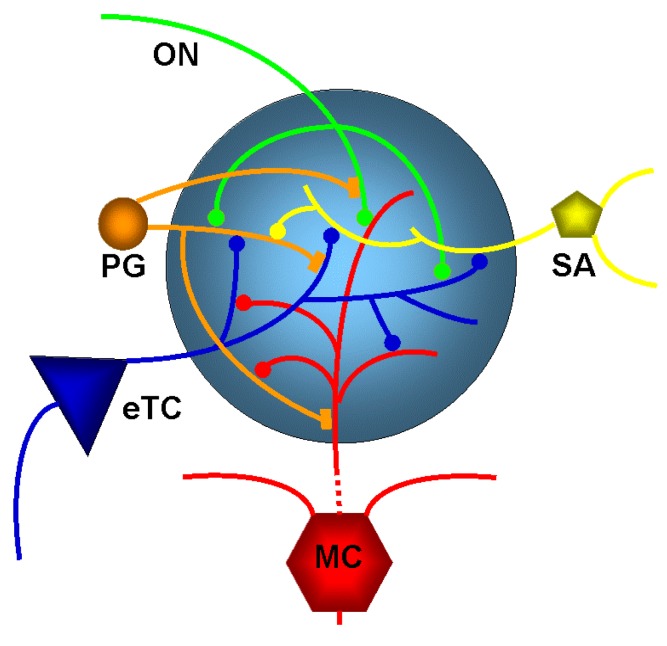
Diagram of the glomerular network. Olfactory nerve (ON) afferents enter the main olfactory bulb through the olfactory nerve laver to synapse with periglomerular cells (PG), mitral cells (MC) and tufted cells (of which only external ones, eTCs are shown) within the glomerular layer. Periglomerular cells inhibit olfactory nerve terminals, external tufted cells and mitral cells. Short Axon (SA) cell axons receive synaptic input from external tufted cells and form extensive interconnections between glomeruli, making glutamate synapses with periglomerular cells, while mitral cell apical dendrites convey sensory information to deeper layers of the main olfactory bulb. Mitral cells and tufted cells form dendrodenritic synapses with periglomerular cells.

The olfactory nerve layer is the gateway for the axons of olfactory receptor neurons to the glomerular layer. It is the home of the olfactory glomeruli which contain axon terminals of olfactory receptor neurons and local interneurons that are collectively referred to as juxtaglomerular (JG) cells (Figure 2). These juxtaglomerular neurons send dendrites into the glomerular neuropil and based on their morphology are referred to external tufted cells, ‘short axon’ cells (which actually have long axons extending through the glomerular layer [18]), and at least two forms of periglomerular cells [19]. Juxtaglomerular neurons communicate extensively with other glomerular neurons (reviewed in [20]). Mitral cells and tufted cells are the output neurons of the main olfactory bulb and send their single apical dendrite into a glomerulus (Figure 2). Olfactory receptor neurons form synapses with mitral and tufted cells and also with juxtaglomerular neurons [7]. Like in other brain systems, glutamate is the major excitatory neurontransmitter in the main olfactory bulb. 

Periglomerular neurons contain dopamine [15,21,22,23] and/or GABA [24] to presynaptically inhibit glutamate release from olfactory nerve terminals. GABA release from periglomerular cells results in feedback GABA_B_ receptor-mediated presynaptic inhibition of olfactory nerve terminals [24,25,26,27] as well as feedforward inhibition via GABA_A_ and GABA_B_ receptor on external tufted cells [28] and other glomerular neurons. A major feature of glomerular circuitry is the reciprocal dendrodendritic synapses formed by periglomerular cells with mitral cells and tufted cells [5,7]. 

External tufted cells rhythmically burst as a result of intrinsic membrane properties [29,30,31]. Furthermore, glutamate that is released from olfactory nerve terminals can function to synchronize the activity of intrinsically bursting external tufted cells. These cells amplify responses to sensory input by synchronous dendritic release of glutamate onto multiple elements within glomeruli, including short-axon cells, periglomerular cells, and mitral cells [29,30,31]. External tufted cells synapse on mitral cells and tufted cells and drive intraglomerular inhibition. This in turn regulates glomerulus output to downstream olfactory networks. The neuronal interactions described above make the glomerulus the site of the first layer of neuromodulation applied to incoming sensory signals from the olfactory receptor neurons. The incoming sensory information is transformed in the main olfactory bulb through different circuit functions such as modification of signal-to-noise ratio, and these modulated signals are conveyed to secondary olfactory structures [1,5].

A second layer of neuromodulation occurs in the deep main olfactory bulb with the input of other interneurons particularly the granule cells, the end result of which might be control over the timing of spiking of mitral cells [1]. The neural circuitry of the main olfactory bulb is therefore highly regulated both by intrinsic neuromodulation from local intrabulbar interneurons (such as periglomerular and short axon cells) as well as extrinsic neuromodulation from cortical inputs and cortical feedback. In fact, there are more cortical (efferent) inputs to the main olfactory bulb than sensory (afferent) inputs and hence the central inputs have strong influence over the processing of olfactory signals [13].

We will now describe the effects seen in the main olfactory bulb resulting from several of the systems that provide neuromodulation.

## 5. Dopaminergic Effects in the Main Olfactory Bulb

Among the juxtaglomerular cells that synapse with olfactory receptor neurons in the glomerular layer of the main olfactory bulb [4], a large subset of the periglomerular cells contains dopamine and/or GABA. Periglomerular cells have been shown to presynaptically inhibit olfactory receptor neurons through dopaminergic and GABAergic transmission (reviewed in [4]). A study by Hsia et al. established that D2 dopamine receptor activation results in a significant depression of synaptic transmission between olfactory receptor neurons and mitral cells [32]. Additionally, endogenous dopamine released in the main olfactory bulb by short-axon cells increases spontaneous bursting frequency of external tufted cells [33]. The short-axon cells co-release GABA and dopamine to evoke a temporally biphasic sequence of inhibition and excitation in external tufted cells. Although the function of dopamine in the olfactory system is not well understood at this time, the relative ease of performing studies in the main olfactory bulb should help us to shed light on some of its other more well-known associations with medical conditions such as Parkinson’s disease, addiction and bipolar disorders. 

## 6. Endocannabinoid Effects in the Main Olfactory Bulb

Endocannabinoids are fatty acid-derived signaling molecules that include *N*-arachidonoylethanolamide (AEA) and 2-arachidonylglycerol (2-AG). They are the endogenous ligands that act at the cannabinoid receptors 1 and 2 and they are generated from membrane lipids when needed at their sites of action. The CB1 receptor in particular (or CB1R) is a 7-transmembrane G protein-coupled receptor, that is widely distributed in the brain and it is involved in a wide range of physiologic activities including learning and memory, feeding behavior, fear, synaptic transmission, synaptic plasticity and growth and development. These receptors are also stimulated by exogenous cannabinoids such as Δ9-tetrahydocannabinol, the bioactive compound in the commonly abused recreational drug marijuana (cannabis), hence the heightened interest in their mechanisms of action [34,35,36].

CB1Rs are expressed in different regions of the brain, including the basal ganglia, cerebellum, hippocampus and olfactory bulb. They are mainly localized at presynaptic terminals where their activation modulate or decrease the release of presynaptic neurotransmitters (such as GABA and Glutamate) via voltage-gated ion channels [36]. Upon their release, endocannabinoids act at CB1Rs on nearby presynaptic terminals to reduce release of neurotransmitters (GABA, glutamate) and, thereby, mediate a form of retrograde signaling. 

Depolarization-induced Suppression of Inhibition (or DSI) is a form of short-term synaptic plasticity mediated by endocannabinoids. In DSI, depolarization of principal neurons triggers the synthesis and release of endocannabinoids which then act on inhibitory interneurons that are presynaptic to the principal neuron to transiently reduce presynaptic firing and neurotransmitter (GABA) release from the interneurons. DSI is considered a mechanism that allows individual neurons to disengage from other cells in their network so they can encode information [36]. The reduced release of the neurotransmitter GABA has been implicated in DSI. A reduction of glutamate release has been shown to play a role in Depolarization-induced Supression of Excitation (DSE), which is also mediated by retrograde action of endocannabinoids and was identified by Kreitzer and Regehr [37] at cerebellar excitatory synapses. DSI and DSE are presynaptic effects as shown by an increase in calcium in the postsynaptic cells and corresponding changes in paired pulse ratio of neurotransmitter release.

Although originally observed in the hippocampus [38] (Wilson and Nicoll, 2000), DSI was found to also occur in the main olfactory bulb [35]. Periglomerular cells express CB1Rs and activation of these receptors inhibits periglomerular cells and reduces their output of inhibitory GABA transmitter. Cannabinoids potently regulate periglomerular cells and external tufted cells, which are two key glomerular neurons. Periglomerular cells form inhibitory GABAergic dendrodendritic synapses with external tufted cells and mitral cells and presynaptically inhibit olfactory receptor neurons. External tufted cells form excitatory glutamatergic dendrodendritic synapses with periglomerular cells. As demonstrated in a brain slice preparation, direct depolarization of external tufted cells evokes suppression of inhibitory postsynaptic current mediated by periglomerular cells in the same external tufted cell. The effect is reversible and can be blocked by receptor blockers of CB1R. The conclusion drawn from this study is that external tufted cells release endocannabinoids which binds to cannabinoid receptors on periglomerular cells [35]. The result is a reduction of GABA release from periglomerular cells which is the demonstration of DSI in the main olfactory bulb and that endocannabinoids modulate glomerular circuit function. Lowered GABA release from periglomerular cells reduces the inhibitory signals to external tufted cells, and in turn reduces inhibition of olfactory receptor neurons and mitral cells. This disinhibition of glomerular cells may serve to make the glomerulus more responsive to sensory inputs [35,36] and may be an important neuromodulatory mechanism in olfactory processing.

Several principles of synaptic processing in the main olfactory bulb have been emerging over the years. One is the dominance of modulatory input from within the main olfactory bulb and through input from higher order olfactory centers by way of centrifugal fibers. Inhibition is a prominent regulator of neural activity in the main olfactory bulb. Inhibitiory synaptic interactions are responsible for shaping synaptic output to cortical areas, and could be regulated by the endocannabinoid system. Inhibitory interactions in the main olfactory bulb manifest themselves through lateral or feedforward inhibition as well as through feedback or recurrent inhibition. Endocannabinoids mediate retrograde signaling in several neural circuits including the main olfactory bulb. Endocannabinoids have been postulated to be critical in regulating centrifugal input to inhibitory granule cells in the main olfactory bulb [39]. Granule cells are inhibitory interneurons and are pivotal in shaping the output of the main olfactory bulb to higher-order olfactory structures [12]. The cell bodies of granule cells are found in the granule cell layer as deep granule cells and also occur more superficially, interspersed with mitral cell somata within the mitral cell layer as superficial granule cells. Granule cells send their apical dendrites to the external plexiform layer and form dendrodendritic synapses with lateral dendrites of mitral and tufted cells. CB1R is strongly expressed in the granule cell layer on axon terminals of centrifugal cortical glutamatergic neurons that project to inhibitory granule cells [39]. While the cellular mechanisms are still under investigation, endocannabinoids are thought to regulate olfactory threshold and food intake. Feeding behavior is a physiologic process that can be regulated by the neuromodulatory effects of endocannabinoids in the olfactory system. In this regard, cannabinoid receptors, CB1Rs were shown by Soria-Gomez et al. to cause fasted mice to increase their feeding behavior (hyperphagia) through an increased detection of food through olfactory mechanisms [39]. The authors performed a wide range of experiments on cannabinoid receptors in the mouse main olfactory bulb and concluded that CB1Rs were expressed in the corticofugal glutamatergic projections to the main olfactory bulb and that activation of CB1R in the main olfactory bulb was the reason of increased feeding to occur after fasting. They further showed that the CB1R control over feeding behavior was by way of the olfactory corticofugal circuits [39]. These important findings linked hunger, olfaction and feeding behavior by a mechanism involving endocannabinoid-mediated neuromodulation of synaptic transmission within the granule cell layer of the main olfactory bulb [39].

Several other recent studies have determined how processing of sensory information in the main olfactory bulb is shaped by massive centrifugal, or feedback, projections from higher cortical areas [40,41]. A study by Pouille and Schoppa [42] examined the role of the endocannabinoid system in regulating centrifugal input to the main olfactory bulb, specifically with respect to cells in the granule cell layer where centrifugal fibers targets both GABAergic granule cells as well as GABAergic deep short-axon cells that inhibit granule cells. Granule cells inhibit mitral cells and tufted cells whereas deep short-axon cells disinhibit mitral cells through inhibiting granule cells. Centrifugal fibers can be either inhibitory or disinhibitory of mitral cells depending on circuit activation. The authors observed that CB1Rs mediate widespread suppressive effects on synaptic transmission at centrifugal fiber synapses onto deep short-axon cell subtypes and centrifugal fiber synapses onto granule cells. As far as the effect on mitral cells is concerned, the authors observed that activation of CB1R could both increase and decrease disynaptic inhibition evoked by centrifugal fiber stimulation. The authors conclude that cannabinoid receptors can bidirectionally change the ratio of inhibition and disinhibition of mitral cells through their effects on centrifugal fibers [42].

The widespread expression of endocannabinoids and their receptors throughout the central nervous system and their distinct effects on neuronal signaling is indicative of an underlying important role for endocannabinoids also in these systems [43].

## 7. Cholinergic Effects in the Main Olfactory Bulb

Acetylcholine (ACh) receptors also play a very active role in the olfactory bulb as cholinergic fibers from the basal forebrain exert strong regulation of olfactory signals and plasticity [5,44,45]. Cholinergic activity is important in regulating the differentiation between similar odors and short-term memory of odors as well as mitral cell tuning curves [46]. 

As a result of neuromodulation from cholinergic inputs, the primary (apical) dendrites of mitral cells are more strongly inhibited by periglomerular cells and the secondary dendrites of mitral cells are more strongly inhibited by granule cells. Mitral cells are made less reactive to weak inputs but remain responsive to stronger signals [4].

Both nicotinic and muscarinic cholinergic receptor subtypes are involved in the mediation of acetylcholine effects in the main olfactory bulb. Neuromodulation involving acetylcholine acting at nicotinic receptors appears to regulate periglomerular cells’ inhibitory circuits that oversee some fine tuning of olfactory sensory perception [6]. Meanwhile, muscarinic agents seem involved in the dense interactions that occur between mitral and granule cells and that constitute the gamma oscillations important for establishing priority among sensory information for processing and for attention [46,47]. The coordinated activation of nicotinic and muscarinic cholinergic receptors appears to tightly regulate the fine tuning of odor representation and also regulate the spike rate and spike timing metrics in the signal processing [46].

Other recent work has determined that muscarinic receptors modulate dendrodendritic inhibitory synapses in the main olfactory bulb to shape output from olfactory glomeruli [48]. Nicotinic (nAChRs) as well as muscarinic acetylcholine receptors (mAChRs) are expressed in olfactory glomeruli. Activation of nAChRs directly excites both mitral cells, tufted cells, and external tufted cells, i.e., the key glomerular output neurons. In contrast, mAChRs activate inhibitory glomerular neurons such as periglomerular cells or short-axon cells as seen as an increase in inhibitory postsynaptic currents at dendrodendritic synapses in response to application of mAChR agonists. Cholinergic projections from the basal forebrain increase the output from the main olfactory bulb by increasing the spike output of mitral and tufted cells [40]. These results underscore the importance of the basal forebrain cholinergic system as a dynamic regulator of the sensitivity to or perceptual quality of odors during active sensing of the olfactory environment [40].

## 8. Serotonergic Effects in the Main Olfactory Bulb

Serotonin is a neuromodulator whose actions are thought to regulate mood and brain states. There is evidence linking disturbances in the serotonergic system with disorders ranging from depression to schizophrenia. Although there is much documentation of the role of serotonin in social stress, anxiety and depression, we know little of how serotonin affects and modulates olfactory behaviors [4,5]. In the main olfactory bulb, serotonin is released by neurons originating in the dorsal and median raphe nucleus. Serotonergic input from the raphe nucleus to the main olfactory bulb targets the glomerular layer, where serotonin (5-HT) acts on periglomerular cells to increase the inhibition of mitral and tufted cells. Serotonin may also synchronize inhibitory inputs among nearby, but not distant pairs of mitral cells, thereby influencing the firing dynamics of the mitral cells. Serotonin indirectly downregulates odor-evoked responses of mitral cells in the main olfactory bulb which emphasizes the importance of centrifugal input in regulating sensory input [49]. Another effect described for serotonin is an excitatory modulation of external tufted cells [50]. This can result in increased external tufted cell excitatory drive on inhibitory neurons (GABAergic/dopaminergic short axon cells and GABAergic periglomerular cells) which increases presynaptic inhibition of olfactory receptor neurons and postsynaptic inhibition of mitral and tufted cells [50]. On the other hand, serotonin not only increases external tufted cell-mediated feedforward excitation onto short-axon cells and periglomerular cells but also onto mitral cells which may enhance mitral sensitivity [51]. Serotonergic afferents from the raphe nucleus dynamically modulate olfactory processing by exerting cell-type specific modulation of olfactory bulb neurons [52].

## 9. Conclusions

The sense of smell relates not only to the identification of odors as being pleasant or unpleasant, but also impacts a wide array of life functions. Recent advances in neurobiology demonstrate that despite its relatively simple organization, the processing within the olfactory system is deceptively complex with its many sources of intrinsic and extrinsic neuromodulation. The dominance of modulatory input is an underlying principle of synaptic processing in the main olfactory bulb, particularly neuromodulatory inhibition as a prominent regulator of neural activity. The endocannabinoid system exemplifies this, in the form of lateral inhibition and recurrent inhibition through retrograde signaling.

Evidence suggests that concurrent activation of different neuromodulatory systems to varying degrees provides multiple and opposing roles on main olfactory bulb neurons transforming sensory signals as they are being routed to secondary structures [5]. It has been postulated that the vast extent of central control over olfactory signal processing in the main olfactory bulb is necessary because these sensory signals are being sent directly to higher cortical centers instead of being passed through several relay stations for sorting and processing as is the case with other senses such as vision or hearing [53].

Altered neuromodulator signaling is evident in most human neurological and psychiatric disorders, including Parkinson’s disease, schizophrenia, depression, and addiction. Because of this, drugs that mimic or block neuromodulators have become important in the treatment of these disorders [43]. Therefore, research into the mechanisms at work in neuromodulation in a relatively simple area such as the olfactory bulb will be invaluable in advancing our understanding of these and other more complicated issues in more complex locations. Hence, additional research will unravel the complexity of signal processing in the main olfactory bulb circuitry and provide great insight into the functioning of the brain as a whole.
